# Polygenic Risk Scores Predicting Estimated GFR Validated With Iohexol Clearance

**DOI:** 10.1016/j.ekir.2025.10.016

**Published:** 2025-10-29

**Authors:** Bjørn O. Eriksen, Matthis Kretzler, Viji Nair, Inger T.T. Enoksen, Stein Hallan, Jon V.N. Porserud, Ludvig Rinde, Toralf Melsom

**Affiliations:** 1 Metabolic and Renal Research Group, UiT The Arctic University of Norway, Tromsø, Norway; 2 Section of Nephrology, Clinic of Internal Medicine, University Hospital of North Norway, Tromsø, Norway; 3 Division of Nephrology, Department of Internal Medicine, University of Michigan, Ann Arbor, Michigan, USA; 4 Department of Computational Medicine and Bioinformatics, University of Michigan, Ann Arbor, Michigan, USA; 5 Department of Nephrology, St Olav’s Hospital, Trondheim, Norway; 6 Department of Clinical and Molecular Medicine, Norwegian University of Science and Technology, Trondheim, Norway

**Keywords:** chronic kidney disease, estimated glomerular filtration rate, measured glomerular filtration rate, genetic expression, glomerular filtration rate

## Abstract

**Introduction:**

Genome-wide association studies (GWAS) have identified hundreds of single nucleotide variants (SNVs) associated with estimated glomerular filtration rate (eGFR). eGFR has been used as a proxy phenotype because of the complexity and cost of measured GFR (mGFR) in large studies. Because eGFR is influenced by non-GFR factors, these GWAS results may be biased compared with a hypothetical study using mGFR. We aimed to investigate this by comparing aggregate measures of genetic effects on mGFR and eGFR.

**Methods:**

We studied 1492 persons from the Renal Iohexol Clearance Survey (RENIS) cohort, a representative sample of the general population in Northern Norway without preexisting cardiovascular disease, kidney disease, or diabetes. We measured iohexol-clearance, and genotyping was performed with a microarray chip enriched for GFR-related SNVs. We compared the performance of 3 published polygenic risk scores (PGS) developed for creatinine-based eGFR (eGFRcr), narrow-sense heritability (h^2^) and the mean effect of SNVs on mGFR, eGFRcr, cystatin C–based eGFR (eGFRcys) and eGFRcr-cys.

**Results:**

The performance of the PGS differed for mGFR and the 3 eGFRs, with best performance for prediction of eGFRcr (*P* < 0.05). However, when the beta coefficients of the SNVs in the 3 PGS were estimated in the RENIS-cohort, their magnitude was 11% to 46% greater for mGFR than for the 3 eGFR methods in 8 of 9 comparisons (*P* < 0.05). mGFR had higher h^2^ (0.47) than eGFRcr (0.21), eGFRcys (0.37), and eGFRcr-cys (0.42).

**Conclusions:**

SNVs with non-GFR effects on creatinine and cystatin-C influence GWAS results. The results of GWAS using eGFR should be validated using experimental and other more precise methods.

## Graphical abstract

**Figure:**
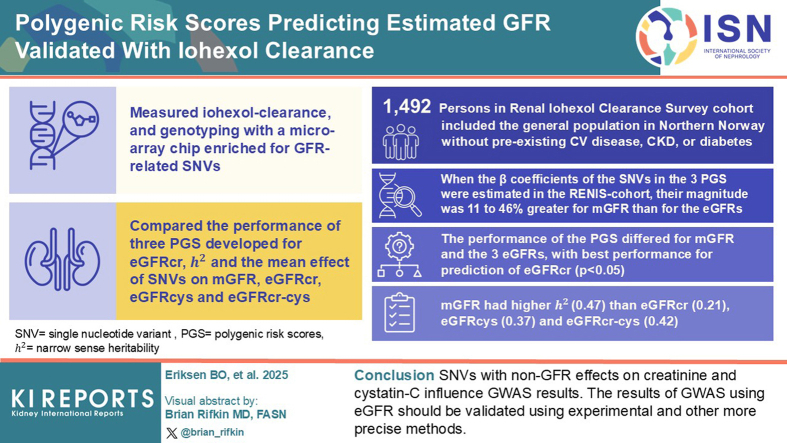

The GFR level is an important risk factor for kidney failure, cardiovascular disease, and death^1^; and varies significantly among individuals^2^, ^3^, ^4^ in the general population. Twin studies indicate that a high percentage of this variation is caused by genetic factors.^5^ GWAS are now the principal method to identify these factors.^6^, ^7^, ^8^, ^9^, ^10^ An understanding of how genes interact with pathogens and environmental factors is necessary to develop effective treatments and preventive measures for acute and chronic kidney disease.

GWAS require very large cohorts to detect genetic variants with small effects.^11^ This constrains the resources available for detailed phenotypic and covariate assessment in each participant. Accordingly, most GWAS of GFR use low-cost eGFRcr as a proxy phenotype for expensive and complicated mGFR. A significant limitation of this method is that serum creatinine is influenced by many well-known non-GFR determinants which may be difficult to discern from genuine GFR associations in a GWAS.^12^, ^13^, ^14^, ^15^ To address this issue, some studies have used blood urea nitrogen and eGFRcys for partial internal validation of findings; however, these markers have typically only been available for a minority of the participants and are influenced by non-GFR determinants.^12^^,^^16^, ^17^, ^18^

To improve the effectiveness of downstream research based on GWAS results of eGFR, an assessment of their overall bias relative to a GWAS of mGFR is important. Although genetic studies with mGFR and sample sizes similar to the 1.5 million persons included in the latest eGFR GWAS are not currently feasible,^9^ comparisons of aggregate measures of genetic effects in smaller study populations are possible.

One such measure is PGS, which quantifies an individual's genetic predisposition to a higher or lower eGFR based on the cumulative effect of multiple genetic variants. PGS are increasingly used for risk stratification and adjustment for genetic effects in kidney research.^19^ However, the use of PGS for kidney function assessed by eGFR presumes their validity relative to mGFR. We aimed to test this assumption in the RENIS cohort. To our knowledge, RENIS is the only population-based study with measurements of iohexol clearance and genotyping with a chip enriched in markers of GFR. Accordingly, we compared PGS across mGFR, eGFRcr, eGFRcys and eGFRcr-cys. We also investigated the heritability of mGFR, and of the bias of the 3 eGFR relative to mGFR.

## Methods

### Study Population

**Figure 1:**
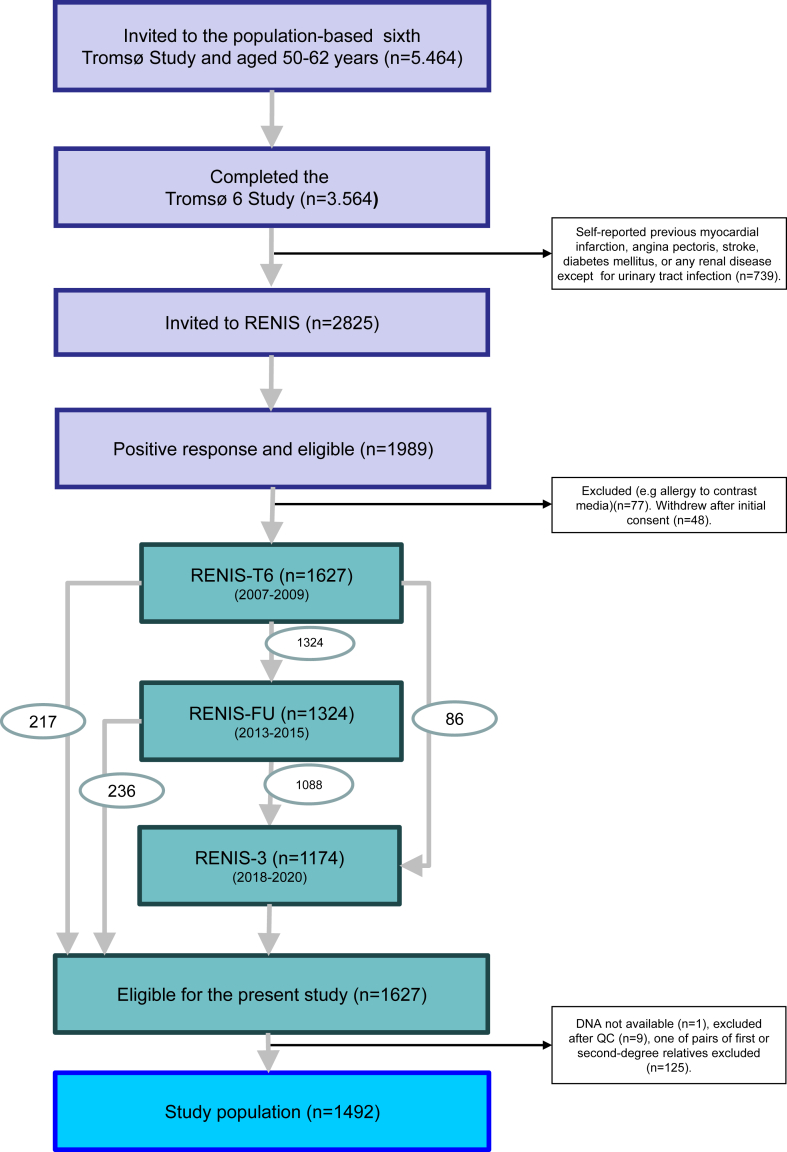
Inclusion of study population from the Tromsø Study prospective population survey. Numbers in oval shapes indicate flow of participants from one round of the study to the next. QC, quality control; RENIS, the Renal Iohexol Clearance Survey.

The RENIS study population is a cohort representative of the general population in the municipality of Tromsø in North Norway. It was recruited in 2007 as a part of the sixth wave of the Tromsø Study, a series of prospective population surveys.^20^ A 40% random sample of individuals aged 50 to 59 years and all individuals aged 60 to 62 years in the municipality were invited to the main part of the Tromsø Study, and 65% participated. Of these, all persons without a self-reported history of previous myocardial infarction, angina pectoris, stroke, diabetes mellitus, or any kidney disease (except urinary tract infection) were invited to RENIS (*N* = 2825). Responders were investigated in random order until a prespecified sample size target of 1627 persons had been included (Figure 1). A total of 1324 and 1174 of the same persons underwent follow-up investigations in 2012 and 2019, respectively. The study population was representative of all eligible subjects and has been described in detail previously.^21^

The study adhered to the principles of the Declaration of Helsinki, and it was approved by the Norwegian Data Inspectorate and the Regional Ethics Committee of North Norway (approval number 89/2006). All subjects provided informed written consent.

### Data

The participants were investigated at the Clinical Research Unit of the University Hospital of North Norway. They answered questionnaires with questions about previous diseases, alcohol use, smoking habits, and current medication. Alcohol use was defined as a dichotomous variable for the weekly use of alcohol or not. Smoking and antihypertensive medication were defined as dichotomous variables for current use.

### Measurement of GFR

In all 3 rounds of RENIS, GFR was measured as single-sample plasma iohexol clearance, which has been validated against gold-standard methods^22^^,^^23^ and has been described in detail previously.^21^^,^^24^ GFR was calculated using Jacobsson’s equations^25^ and standardized to 1.73 m^2^ of body surface area. Body surface area was estimated using the equation of DuBois and DuBois.^26^

### Other Measurements

#### Blood Pressure

Attended office blood pressure was measured after 2 minutes of rest in the seated position with an automated device (model UA 799; A&D, Tokyo, Japan) by a study nurse.^27^ Hypertension was defined as office systolic blood pressure ≥ 140 mm Hg or office diastolic blood pressure ≥ 90 mm Hg or the use of antihypertensive medication according to the guidelines of the European Society of Hypertension.^28^

#### Biochemical Analyses

Fasting serum glucose, triglycerides, and low-density lipoprotein cholesterol and high-density lipoprotein cholesterol were measured with standard methods as described previously.^27^ Serum creatinine was measured using an enzymatic assay standardized to the isotope dilution mass spectrometry method (CREA Plus, Roche Diagnostics, GmbH, Mannheim, Germany). Cystatin C was measured using a particle-enhanced turbidimetric immunoassay (Gentian, Moss, Norway) calibrated to the international reference ERM-DA471/IFCC.^29^ High-sensitivity C-reactive protein and glycated hemoglobin were measured in the main sixth Tromsø Study, as described previously.^30^^,^^31^ Diabetes mellitus was defined as fasting glucose ≥ 7.0 mmol/l or glycated hemoglobin ≥ 6.5% (48 mmol/mol). In accordance with the inclusion criteria, none of the included participants used antidiabetic medication at baseline.

#### Estimated GFR

eGFR was calculated using the original Chronic Kidney Disease Epidemiology Collaboration equations published in 2009 and 2012 (eGFRcr, eGFRcys, and eGFRcr-cys),^32^,^33^ which have been shown to perform slightly better in a North European population^34^^,^^35^ than the new race-free versions.

### Phenotypes

#### Definitions

The phenotypes used in this study were derived from the individual levels of the 4 GFR assessment methods mGFR, eGFRcr, eGFRcys and eGFRcr-cys, which we will refer to as the “GFR phenotypes.” In addition, we defined the 3 differences between mGFR and each of the 3 eGFR as phenotypes (mGFR − eGFRcr, mGFR – eGFRcys, and mGFR − eGFRcr-cys). These 3 differences represent the bias of each eGFR relative to mGFR and will be referred to as the “bias phenotypes.” The bias phenotypes were defined to allow the estimation of their heritability.

### Statistical Methods for Estimating Phenotypes

We estimated each of these 7 phenotypes as the individual random intercepts in separate mixed models that included all repeated measurements for each person.^36^ This contributes to minimizing random error and maximizing statistical power as opposed to relying on baseline measurements alone.^37^

We used the general additive version of mixed models (GAMM) found in the mgcv-package in R.^38^ A GAMM with separate nonlinear slopes for women and men and adjustment for baseline age and health status has previously been found to provide the best fit to the longitudinal GFR assessments in RENIS.^21^ In the present investigation, we used the same model without health adjustment to avoid blocking genetic effects mediated by comorbid conditions and risk factors.

As dependent variables in separate GAMMs we used the 4 GFR assessment methods (mGFR, eGFRcr, eGFRcys, and eGFRcr-cys) standardized to 0 mean and unit variance, and the 3 biases calculated as the differences between these standardized mGFR and eGFR. We used observation time since baseline as the time variable and adjusted for the first 10 principal components and their interactions with time. The model used an unstructured covariance matrix. In each of the 7 models, the best linear unbiased predictors of the random intercept adjusted to the population mean age were taken to represent the corresponding phenotype for each individual. There was a large improvement in the Akaike Information Criterion for the GAMMs of the bias phenotypes when random effects were included. This demonstrates that the biases are persistent traits that can be estimated for each individual and not mere measurement errors relative to mGFR (Supplementary Table S1). This provides the rationale for defining the biases as phenotypes.

For each phenotype, the distribution of residuals in the linear regressions with the PGS was checked visually with and without log-transformation (Supplementary Table S2 and Figure S1). Log -transformation of all the phenotypes resulted in more skewed residuals than without transformation (Supplementary Figure S1). Accordingly, unlike most genetic studies of GFR, we did not use log-transformation of the phenotypes for the analyzes in this investigation.

### Genotyping

#### Isolation of DNA

Samples of whole blood were obtained from all the 1627 participants in the RENIS cohort at baseline between the November 14, 2007 and June 10, 2009. DNA was extracted automatically with kits from Perkin Elmer on the Hamilton Chemagic Star instrument. A quality check of DNA yield was performed with NanoDrop One.

#### Customization of Microarray

Genotyping was performed by the Human Genomics Facility of the Genetic Laboratory of the Department of Internal Medicine at Erasmus MC, Rotterdam. We used a custom version of the Illumina Infinium Global Screening Array-24+ v3.0 Beadchip (https://emea.illumina.com/) enriched with markers of SNVs found to be associated with eGFRcr in the study by Stanzick *et al.*, which was the largest GWAS available at the time of study planning.^8^

#### Quality Control

Quality control of the genotyping was performed by The Human Genomics Facility of the Genetic Laboratory of the Department of Internal Medicine at Erasmus MC.

Five samples and 4509 SNVs were removed because of call rates < 97.5%. In the Hardy-Weinberg equilibrium test, 102 SNVs were excluded from the dataset because of being out of Hardy-Weinberg equilibrium (*P* < 1∗10^-5^). Individual heterozygosity tests were conducted using PLINK.^39^ No samples with evidence for excess of heterozygosity were identified. Eighteen samples with evidence for excess of homozygosity were identified but were not excluded.

A check for sex mismatches using the sex-variable in the RENIS dataset and the PLINK call of gender from the X-chromosome was performed, but no mismatch was detected.

After the primary quality control, zCall was used to detect previously uncalled genotypes, mostly of rare SNVs.^40^

Genetic ancestry was investigated using PLINK by calculating identity by state or identity by descent distances between individuals and clustering with reference to the 2504 samples of the 1000 Genomes Phase3v5 reference dataset.^41^ In total, there were 8 samples with non-European genetic ancestry, which were kept in the dataset.

Cryptic family relationship within the study population were identified by calculating pairwise identity by descent distances using KING software. A total of 125 pairs of samples were detected with expected first or second-degree familial relationships.

More details about the genotyping of the RENIS cohort can be found in the Supplementary Methods.

### Imputation

After quality control, 640,035 SNVs in 1617 persons were available for imputation, which was also performed by The Human Genomics Facility of the Genetic Laboratory of the Department of Internal Medicine at Erasmus MC. The Haplotype Reference Consortium (HRC) panel version 1.1 (HRC r1.1) was used as reference panel.^42^ Imputation was done using SHAPEIT^43^ and Minimac 4^44^ for the phasing and imputation, respectively. SNVs with MACH R^2^ > 0.8 were used for analyses in the present study. Only SNVs on autosomal chromosomes were considered. Additional details about the imputation can be found in the Supplementary Methods.

### PGS

#### Selection of PGS

An individual’s PGS is the weighted sum of the number of risk alleles across SNVs found to influence the eGFR level in a GWAS. The weights are typically beta coefficients obtained from the regression analyses of the eGFR on the number of risk alleles at each SNV.^19^

We searched the PGS catalogue with the search terms “GFR,” “eGFR,” or “CKD” on November 23, 2023.(https://www.pgscatalog.org/) Thirteen publications were identified.^6^^,^^7^^,^^10^^,^^45^, ^46^, ^47^, ^48^, ^49^, ^50^, ^51^, ^52^, ^53^, ^54^ An additional PGS was developed by Stanzick *et al.*, but was not found in the PGS catalogue.^8^ Three of these 14 publications did not develop new PGS^52^^,^^53^ or included only patients with diabetes^51^ and were not considered further. Two publications from the UK Biobank had the same PGS.^47^^,^^48^ All the publications used data from the CKDGen Consortium^6^, ^7^, ^8^^,^^45^^,^^46^^,^^50^^,^^54^ and/or the UK Biobank,^6^, ^7^, ^8^^,^^10^^,^^48^ either partly or wholly for discovery or validation.

To evaluate PGS with different characteristics, we chose the study by Khan *et al.* (PGS catalog ID PGS002237), the most recent study that was mainly based on CKDGen consortium GWAS summary statistic^6^; and the study by Sinnott-Armstrong *et al.* (PGS catalog ID PGS000682), which was mainly based on UK Biobank data.^47^ In addition, we evaluated the PGS of Stanzick *et al.* (PGS_Stanzick), which used GWAS data from both of these sources.^8^ Whereas the PGS000682 and PGS_Stanzick were developed with eGFRcr as the continuous phenotype variable, the PGS002237 used a dichotomous CKD phenotype defined as eGFRcr < 60 ml/min per 1.73 m^2^. All three phenotypes were assumed to represent the same underlying genetic determinants of GFR.

#### Calculation of PGS for the Participants in the RENIS Cohort

The PGS of the PGS000682 and the PGS002237 were downloaded from the PGS catalogue ((https://www.pgscatalog.org/). The PGS_Stanzick was extracted from the results for the 634 independent index lead variants in Supplementary Data 6 of her publication.^8^ We used the beta-coefficients for log(eGFRcr) given in this table as weights for the PGS, although the paper describes using beta-coefficients for untransformed eGFRcr in its PGS analyses. Because the correlation between these 2 sets of coefficients will be very high, this makes little difference in the results. A total of 320 (75%) of the lead variants of Stanzick’s 424 eGFRcr loci were supported by results for eGFRcys.^8^

The scores for PGS002237, which was developed to indicate risk for eGFRcr < 60 ml/min per 1.73 m^2^, have been multiplied by −1 to correspond to the other 2 PGS, where an increasing score indicates risk for higher GFR. Of the 471,316 variants included in the score, only the 41,426 variants with non-0 weights were included in this investigation. We did not include the SNVs in mitochondria or on sex chromosomes in PGS000682.

Weighted PGS for each individual for each PGS was calculated as the sum of dosage multiplied by the published variant weights using the score option in PLINK 2.0.

#### Validation of PGS

We performed separate multiple linear regression analyses with each of the 4 GFR phenotypes as the dependent variable and the weighted PGS as the independent variable. The PGS independent variables were scaled to 0 mean and unit variance. Separate regressions were performed for each of the 3 PGS. We adjusted for baseline age, biological sex, and the 10 first principal components. The percentage of phenotype variation explained by each PGS was estimated as the square of the beta coefficient for the PGS multiplied by the ratio between the variance of the PGS and the variance of the phenotype. The hypotheses of no difference between the mGFR and each of the 3 eGFR phenotypes were tested using the bootstrap method from 2000 resamples from the study population. We used the adjusted bootstrap percentile method with the boot-package in R and set the alpha of the test at 0.05.^55^

### Comparison of Individual SNV Effects With Deming Regression

To compare the magnitude of the effects of the individual SNVs included in the 3 PGS between the mGFR and the eGFR phenotypes, we used the same linear regression models with the individual SNVs as independent variables. This estimated the effect of each marker on the GFR phenotypes. For each of the 3 comparisons between mGFR and eGFR, we then compared the marker effects in Deming regression models. Deming regression gives unbiased estimates of the association between 2 methods that are both affected by measurement error.^56^ The ratio between the variances of measurement error of the 2 phenotypes, which is a necessary parameter in Deming regression, was set at unity. The degree of association between the effect estimates of 2 phenotypes was judged from the slope of the regression line between them. The mcr-package in R was used for these comparisons (https://CRAN.R-project.org/package=mcr).

### h^2^

We used Genome-wide Complex Trait Analysis software to estimate the h^2^ for the phenotypes.^57^ The h^2^ estimates the proportion of phenotypic variance caused by the variation in the SNVs. When constructing the genetic relationship matrix, SNVs with a minor allele frequency < 1% were excluded. One of a pair of individuals with estimated relatedness > 0.025 was removed before estimating h^2^ with the Generalized Restricted Estimated Maximum Likelihood approach.^58^ Sex, age at baseline, and the first 10 principal components were used as covariates in these analyses. h^2^ was estimated for the 4 GFR phenotypes and the 3 bias phenotypes.

### Statistical Methods

The analyses performed in this study were done with R version 4.3.2 (2023-10-31 ucrt) unless otherwise specified.^59^ Statistical significance was set at *P* < 0.05.

## Results

### Study Population

DNA was available from 1626 of the 1627 persons included in the baseline RENIS cohort. After quality control and imputation, there were 11,980,069 SNVs available for 1617 individuals. One random person from each pair of 125 samples with expected first or second-degree familial relationships was excluded, resulting in a study population of 1492 participants with a total of 3860 GFR measurements over a median follow-up of 11 years (Figure 1).

The baseline characteristics of the study population according to sex are shown in Table 1. There were 761 women (51%) and 731 men (49%), both with a mean age of 58 years. A total of 638 persons (43%) had hypertension. Although persons with self-reported diabetes had been excluded, 28 persons (1.8%) met the criteria for diabetes based on fasting glucose or glycated hemoglobin. The mean baseline mGFR was 94 ml/min per 1.73 m^2^. Sex differences and the differences between mGFR and eGFR have been discussed in previous publications.^14^^,^^15^^,^^21^^,^^29^^,^^60^^,^^61^

**Table 1:** Study population baseline characteristics

Characteristics	Women (*n* = 761)		Men (*n* = 731)		All (*N* = 1492)	
Age, yrs	58.1	(3.9)	58.0	(3.8)	58.0	(3.8)
Height, cm	164.5	(6.0)	177.0	(6.2)	170.6	(8.7)
Body weight, kg	72.6	(12.4)	87.4	(12.4)	79.8	(14.4)
Body mass index, kg/m^2^	26.8	(4.4)	27.9	(3.5)	27.3	(4.0)
Total cholesterol, mg/dl	5.7	(0.9)	5.6	(1.0)	5.6	(0.9)
LDL cholesterol, mmol/l	3.6	(0.9)	3.7	(0.8)	3.7	(0.9)
HDL cholesteroll, mmol/l	1.7	(0.4)	1.4	(0.4)	1.5	(0.4)
Fasting triglycerides, mmol/l	1.0	(0.7–1.3)	1.1	(0.8–1.6)	1.0	(0.8–1.5)
Lipid-lowering medication, *n* (%)	56	(7 %)	46	(6%)	102	(7%)
Current smoker, *n* (%)	176	(23 %)	141	(19 %)	317	(21%)
Use of alcohol at least weekly, *n* (%)	201	(27 %)	213	(29 %)	414	(28%)
Systolic blood pressure, mm Hg	125.7	(17.4)	134.1	(16.9)	129.8	(17.6)
Diastolic blood pressure, mm Hg	80.9	(9.3)	86.2	(9.3)	83.5	(9.7)
Hypertension, *n* (%)	268	(35 %)	370	(51 %)	638	(43 %)
Antihypertensive medication, *n* (%)	132	(17 %)	138	(19 %)	270	(18 %)
Fasting glucose, mmol/l	5.1	(4.9–5.4)	5.4	(5.2–5.8)	5.3	(5.0–5.6)
Hemoglobin A1c, %	5.5	0.4	5.6	0.4	5.6	0.4
Diabetes, *n* (%)	9	(1 %)	19	(3 %)	28	(2%)
High sensitivity CRP, mg/l	2.3	(3.9)	2.8	(8.5)	2.5	(6.6)
Absolute measured GFR, ml/min	92.8	(15.3)	115.7	(17.7)	104.0	(20.1)
Measured GFR (mGFR), ml/min per 1.73 m^2^	89.9	(13.9)	98.0	(13.5)	93.9	(14.3)
Creatinine-based estimated GFR						
(eGFRcr)^a^, ml/min per 1.73 m^2^	94.6	(9.8)	95.4	(8.8)	95.0	(9.3)
Cystatin C-based estimated GFR						
(eGFRcys)^a^, ml/min per 1.73 m^2^	102.2	(12.0)	108.6	(11.6)	105.3	(12.2)
Creatinine and cystatin C-based estimated GFR						
(eGFRcr-cys)^a^, ml/min per 1.73 m^2^	101.4	(11.7)	104.7	(10.5)	103.0	(11.3)

CRP, C-reactive protein; eGFR, estimated glomerular filtration rate; HDL, high-density lipoprotein; LDL, low-density lipoprotein; RENIS, the Renal Iohexol Clearance Survey.

Estimates are given as mean (SD), median (interquartile range) or *n* (percent).

a GFR estimated with equations published by the Chronic Kidney Disease Epidemiology Collaboration. ^32^^,^^33^

### PGS

The means (SD) of the PGS according to sex are shown in Table 2. The mean scores for men and women were nearly identical. The increase in each of the 4 GFR phenotypes per 1 SD increase in each of the weighted PGS is shown in Table 3. The results for each GFR phenotype were very similar across the 3 PGS, but there were differences between the phenotypes within each PGS. The increase in the mGFR phenotype varied from 0.13 to 0.15, which was less than that for the eGFRcr phenotype for all the 3 PGS (*P* < 0.05), but greater than that for eGFRcys (*P* < 0.05), except for PGS000682. The percentage of phenotypic variation explained by the PGSs varied between 3.6 and 4.4 for mGFR but was consistently higher for both the eGFRcr and eGFRcr-cys phenotypes (Table 3).

**Table 2:** Polygenic risk scores

PGS	Number of SNVs in PGS	Number of SNVs available in RENIS (%)		Score type	Women (*n* = 761)		Men (*n* = 731)		All (*N* = 1492)		Reference
PGS_Stanzick	634	604	(95)	Unweighted	629	(14)	628	(14)	629	(14)	8
				Weighted	−1.76	(0.04)	−1.75	(0.04)	−1.76	(0.04)	
PGS002237^a^	41426	41024	(99)	Unweighted	64490	(132)	64486	(133)	64488	(132)	6
				Weighted	−0.68	(0.29)	−0.69	(0.28)	−0.68	(0.28)	
PGS000682^b^	17467	16229	(93)	Unweighted	19160	(87)	19160	(85)	19160	(86)	47
				Weighted	−0.17	(0.04)	−0.17	(0.04)	−0.17	(0.04)	

PGS, polygenic risk score; RENIS, the Renal Iohexol Clearance Survey; SNV, single nucleotide variant.

Scores are given as mean (SD) and were calculated as the sum of genetic dosage for each SNV with or without multiplication by weight.

a Of the 471316 SNVs included in this PGS, only those with non-0 weights were considered in this investigation. Weights were multiplied by −1 to indicate increasing GFR instead of odds ratio for GFR < 60 ml/min per 1.73 m2.

b SNVs on sex chromosomes and in mitochondria were not considered.

**Table 3:** Associations of PGS with GFR phenotypes^a^ in multiple linear regression

Phenotype^a^	PGS	Beta per 1 SD increase in PGS (95% confidence interval)		*P*	Phenotypic variance explained
mGFR	PGS_Stanzick	0.15	(0.11–0.18)	5.23E-16	4.4%
	PGS002237	0.13	(0.10–0.17)	1.06E-12	3.6%
	PGS000682	0.14	(0.11–0.18)	3.74E-15	4.2%
eGFRcr	PGS_Stanzick	0.19^b^	(0.15–0.22)	2.05E-29	8.4%
	PGS002237	0.17^b^	(0.14–0.21)	2.42E-24	7.1%
	PGS000682	0.20^b^	(0.17–0.23)	1.95E-34	9.8%
eGFRcys	PGS_Stanzick	0.11^b^	(0.08–0.13)	4.05E-14	3.9%
	PGS002237	0.09^b^	(0.06–0.12)	1.90E-10	2.9%
	PGS000682	0.13	(0.11–0.16)	3.93E-21	5.9%
eGFRcr-cys	PGS_Stanzick	0.17	(0.14–0.20)	1.49E-26	7.5%
	PGS002237	0.15	(0.12–0.18)	4.70E-21	6.2%
	PGS000682	0.19	(0.16–0.22)	4.35E-35	10.0%

eGFR, estimated GFR; GFR, glomerular filtration rate; mGFR, measured GFR; PGS, polygenic risk score; RENIS, the Renal Iohexol Clearance Survey.

Each line represent a separate multiple linear regression with GFR phenotype as dependent variables, standardized weighted PGS as independent variables and adjustments for age, sex and the first ten principal components.

a Each phenotype was calculated with a generalized additive mixed model after standardization to 0 mean and unit variance. See Methods.

b P < 0.05 for difference from mGFR with the same PGS as assessed with the bootstrap method.

We estimated the effects of the SNVs included in the 3 PGS for the participants in the RENIS cohort and compared the effects across the GFR phenotypes with Deming regression. The slopes of the regressions give an overall estimate of the relative magnitude of the effect sizes (Table 4). The slopes for mGFR relative to eGFRcr ranged from 1.07 to 1.13, and for mGFR relative to eGFRcys from 1.40 to 1.46, demonstrating stronger associations between the SNVs and the mGFR than the eGFR phenotypes. Except for the eGFRcr phenotype for PGS_Stanzick, these slopes were all statistically significantly different from unity (*P* < 0.05).

**Table 4:** Deming regression for comparison of the associations of the SNVs included in the polygenic risk scores between the GFR phenotypes

		X-variable					
		eGFRcr phenotype		eGFRcys phenotype		eGFRcr-cys phenotype	
Y-variable	Polygenic risk score	Beta (95% confidence interval)		Beta (95% confidence interval)		Beta (95% confidence interval)	
mGFR phenotype	PGS_Stanzick	1.07	(0.97–1.16)	1.40	(1.27–1.53)	1.11	(1.03–1.19)
	PGS002237	1.13	(1.12–1.15)	1.46	(1.44–1.48)	1.18	(1.17–1.19)
	PGS000682	1.11	(1.09–1.13)	1.45	(1.42–1.48)	1.17	(1.15–1.19)

eGFR, estimated GFR; GFR, glomerular filtration rate; mGFR, measured GFR; PGS, polygenic risk score; RENIS, the Renal Iohexol Clearance Survey; SNV, single nucleotide variant.

For each PGS and combination of the mGFR phenotype as Y-variable and either of the eGFR phenotypes as X-variable, each line presents the beta (slope) in a Deming regression comparing SNV-associatons included in the corresponding risk score. The absolute value of the intercepts in all these Deming regressions were estimated at < 0.004. A beta > 1 indicates an overall stronger association between the SNVs in the PGS and the mGFR phenotype than with the X-variable.

### h^2^

The estimates of h^2^ are based on all the genotyped and imputed SNVs in the cohort, not just those included in the PGS. The results for the 4 GFR phenotypes are shown in Table 5. Forty-seven percent of the variability in the mGFR phenotype could be explained by variation in the SNVs, which was substantially higher than for both eGFRcr (21%) and eGFRcys (32%). However, the h^2^ for eGFRcr-cys (42%) was just slightly lower than for mGFR. Bias for eGFRcys and eGFRcr-cys had a substantial h^2^ (0.56 and 0.48) (*P* < 0.05 for the difference from 0), whereas the h^2^ for eGFRcr bias was low and not statistically significantly different from 0 (*P* = 0.50).

**Table 5:** Narrow-sense heritability for the GFR and bias phenotypes

Phenotype	Genetic variance, V(G)		Phenotypic variance, V(p)		Heritability, h^2^ = V(G)/V(p)		Residual variance, V(e)		*P*-value for h^2^ different from 0
GFR phenotypes									
mGFR	0.23	(0.09)	0.48	(0.02)	0.47	(0.19)	0.25	(0.09)	0.005
eGFRcr	0.09	(0.08)	0.41	(0.02)	0.21	(0.19)	0.32	(0.08)	0.14
eGFRcys	0.11	(0.06)	0.30	(0.01)	0.37	(0.19)	0.19	(0.06)	0.02
eGFRcr-cys	0.16	(0.07)	0.38	(0.01)	0.42	(0.19)	0.22	(0.07)	0.009
Bias phenotypes									
mGFR−eGFRcr	0.02	(0.06)	0.30	(0.01)	0.08	(0.18)	0.28	(0.06)	0.50
mGFR−eGFRcys	0.14	(0.05)	0.24	(0.01)	0.56	(0.19)	0.11	(0.05)	0.001
mGFR−eGFRcr-cys	0.05	(0.02)	0.11	(0.00)	0.48	(0.19)	0.06	(0.02)	0.005

eGFR, estimated GFR; GFR, glomerular filtration rate; mGFR, measured glomerular filtration rate.

Results are given as estimate (standard error).

The GFR phenotypes were standardized to 0 mean and unit variance and calculated with a generalized additive mixed model. The bias phenotypes are the differences between the standardized mGFR and the standardized eGFR and also calculated with generalized additive mixed models. See Methods.

## Discussion

To our knowledge, this is the first study where genetic determinants of eGFR and mGFR have been compared. Because there are clear biochemical and physiological differences between creatinine (a metabolite), iohexol (an exogenous chemical compound) and cystatin C (a protein enzyme inhibitor), differences in their genetics would be expected. Accordingly, we found the results for mGFR and eGFRcys nearly identical across the tested PGS, but consistently inferior to those for eGFRcr and eGFRcr-cys (Table 3).

The finding of a substantially lower percentage of variance explained for the mGFR than for the eGFRcr standardized phenotypes within each PGS suggests that all 3 PGS include SNVs associated with non-GFR determinants of serum creatinine. The similar finding of a lower percentage for eGFRcys supports this explanation. Yu *et al.* also found the same relationship between variance explained for eGFRcr and eGFRcys with a PGS developed for eGFRcr.^7^

In contrast to the PGS, the h^2^ estimates the proportion of phenotype variability explained by all the genotyped and imputed SNVs. h^2^ was 0.49 for mGFR in this study, which is of the same magnitude as for eGFRcr in twin studies summarized by Jefferis *et al.* (range: 0.33–0.78),^62^ but somewhat higher than for eGFRcr in GWAS (range: 0.08–0.27).^5^^,^^47^^,^^63^^,^^64^ The h^2^ for eGFRcr (0.21) had less than half the heritability of mGFR (Table 5). This suggests that many SNVs with effects on mGFR will not be included in a PGS developed with eGFRcr as phenotype or that their effects will be weaker. The h^2^ for eGFRcys and eGFRcr-cys (0.37 and 0.42) were of a similar magnitude to that of mGFR. This could reflect either that they are better estimators of mGFR than eGFRcr, or that the h^2^ is high because of non-GFR genetic determinants of cystatin-C. The finding that both eGFRcys bias and eGFRcr-cys bias have high h^2^ (0.56 and 0.48) (Table 5) favors the last explanation.

When recalculating the beta-coefficients for the individual SNVs included in the 3 PGS in the study population, the coefficients for mGFR were overall higher than for the 3 eGFRs (Table 4). This indicates that a PGS developed with mGFR as phenotype could perform better than the 3 examined PGS. Because of the limited size of the study cohort and the lack of cohorts with mGFR for external validation, we did not construct an mGFR PGS for this investigation.

The main strength of our investigation was its population-based prospective design with repeated measurements of GFR according to a validated method. To our knowledge, the RENIS cohort is unique in this respect. It is independent of the CKDGen Consortium and UK Biobank, which have been used for the development and/or validation of the majority of PGS. In addition, the enrichment of the microarray chip with SNVs from previous GWAS resulted in high-quality genotype data at the individual level, as opposed to summary-level data used in meta-analyses.

The most important limitation of the present study was low statistical power to characterize the bias of eGFR at the SNV, gene or pathway level. In addition, this limited the precision of the heritability estimates, which should be interpreted with caution. Because the majority of the participants in RENIS have > 1 GFR measurement, we were able to reduce random error and increase statistical power by using a GAMM that incorporated all the mGFRs for each individual. We included only middle-aged persons with European ancestry, which limits the generalizability to other age-groups or ancestries. However, we believe that the principled relationships between genes and GFR change little with age, although the genetic effects may diminish.^62^ We excluded persons with diabetes, CVD and kidney disease, which implies that our findings could have been different in a population of patients. A low prevalence of risk factors for large discrepancies between eGFR and mGFR such as sarcopenia, poor nutrition, and inflammatory conditions in this community dwelling population may have affected the results for the bias phenotypes relative to study populations with more comorbidity.

We conclude that PGS developed from eGFRcr exhibit inferior performance in external validation when mGFR is used as the phenotype. The performance further deteriorates with eGFRcys as the phenotype. The influence of SNVs on bias is more substantial for eGFRcys than for eGFRcr. For this reason, eGFRcr is a better choice than eGFRcys as a proxy phenotype for mGFR in large GWAS. The applicability of eGFRcr-cys may be constrained by its dependence on non-GFR genetic effects on both markers. These limitations suggest that candidate genetic markers from GWAS of eGFR require validation through experimental or other methods.

## Disclosure

MK reports support from the NIH/NIDDK; funding through the University of Michigan from the Chan Zuckerberg Initiative, Breakthrough T1D, Alport Foundation, Roche-Genentech, AstraZeneca, Novo Nordisk, Moderna, European Union Innovative Medicine Initiative, Boehringer-Ingelheim, Certa, Travere Therapeutics, Maze therapeutics, Chinook, RenalytixAI, Eli Lilly, Gilead, Regeneron, IONIS Pharmaceuticals, Jansen and Sanofi; consulting fees through the University of Michigan from Janssen, Novo Nordisk, Otsuka, Alexion, Variant Bio, and Novartis; and serves on the American Society of Nephrology Program committee and the Nephcure Kidney International Board. MK and VN have a patent— PCT/EP2014/073413 “Biomarkers and methods for progression prediction for chronic kidney disease”—licensed. All the other authors declared no competing interests.

### Funding

This study was funded by the North Norwegian Regional Health Authority, which had no role in the design and conduct of the study or the collection, management, analysis, and interpretation of the data, review or approval of the manuscript for submission.

## Data Availability Statement

The data underlying this article cannot be shared publicly because this was not included in the research permission due to ethical considerations and the privacy of individuals who participated in the study. The data can be shared on request as part of a research collaboration.Please contact the corresponding author, BOE (bjorn.odvar.eriksen@unn.no) or the last author, TM (toralf.melsom@unn.no).

## Supplementary Material

Supplementary File (PDF) **Supplementary Methods. Supplementary References. Figure S1.** Q-Q plots for original and log-transformed versions of the GFR phenotypes. **Figure S2.** Principal component analysis. **Table S1.** Akaike Information Criterion for GAMMs of bias phenotypes with and without random effects. The Renal Iohexol Clearance Survey (RENIS). **Table S2.** Shapiro-Wilk tests of normality for original and log-transformed GFR phenotypes. The Renal Iohexol Clearance Survey (RENIS).STROBE statement.
