# Supplementary material for: Polygenic Risk Scores Predicting Estimated GFR Validated With Iohexol Clearance
Source: Kidney Int Rep. 2025 Oct 29;11(1):196–206. doi: 10.1016/j.ekir.2025.10.016 (PMC12799510; doi:10.1016/j.ekir.2025.10.016)
Supplement: Supplementary File (PDF) — Supplementary Methods. Supplementary References. Figure S1. Q-Q plots for original and log-transformed versions of the GFR phenotypes. Figure S2. Principal component analysis. Table S1. Akaike Information Criterion for GAMMs of bias phenotypes with and without random effects. The Renal Iohexol Clearance Survey (RENIS). Table S2. Shapiro-Wilk tests of normality for original and log-transformed GFR phenotypes. The Renal Iohexol Clearance Survey (RENIS).STROBE statement. [file mmc1.pdf]

# Supplementary Material

# Polygenic risk scores predicting estimated GFR validated with iohexol clearance

Bjørn O. Eriksen,[1,2] M.D. Ph.D., Matthis Kretzler[3,4] M.D. Ph.D., Viji Nair[3] M.S., Inger T. T. Enoksen[1] M.D. Ph.D., Stein Hallan[5,6] M.D. Ph.D., Jon V. N. Porserud[1,2] M.D. Ph.D., Ludvig Rinde[1,2] M.D. Ph.D., Toralf Melsom[1,2] M.D. Ph.D.

[1]Metabolic and Renal Research Group, UiT The Arctic University of Norway, Tromsø, Norway

[2]Section of Nephrology, Clinic of Internal Medicine, University Hospital of North Norway, Tromsø, Norway

[3]Division of Nephrology, Department of Internal Medicine, University of Michigan, Ann Arbor, MI, USA

[4]Department of Computational Medicine and Bioinformatics, University of Michigan, Ann Arbor, MI, USA

[5]Department of Nephrology, St Olav's Hospital, Trondheim, Norway

[6]Department of Clinical and Molecular Medicine, Norwegian University of Science and Technology, Trondheim, Norway

Corresponding author: Bjørn Odvar Eriksen; Phone: +47 466 82 780; e-mail: bjorn.odvar.eriksen@unn.no; mailing address: Sykehusveien 38, 9038 Tromsø, Norway

# Supplementary Methods

## Genotyping

### Isolation of DNA

Samples of whole blood were obtained in EDTA 6 mL tubes and immediately frozen at -20 °C and transferred to storage at -80 °C within a few days. The samples were retrieved and shipped to the Helseundersøkelsen i Nord-Trøndelag (HUNT) Biobank on dry ice on the 28th of September 2021.

At HUNT Biobank, DNA was extracted automatically on the Hamilton Chemagic Star instrument. A quality check of DNA yield was performed of every eighth sample. The DNA concentration was measured with PicoGreen in all samples. The samples were diluted and normalized to the required concentration and volume with the Biomek i5s8. A DNA integrity test was run on 5% of the samples (n=96) using the Tapestation instrument with median result 9.0 DIN (range 7.6 – 9.5 DIN).

The DNA samples were shipped on dry ice to The Human Genomics Facility of the Genetic Laboratory of the Department of Internal Medicine at Erasmus MC, Rotterdam, on the 27th of June 2022. Two hundred nanograms of DNA for each participant were sent in 96 well Abgene Storage Plates. Four microliters of 50 ng/µL were sent for most of the samples, but 8 µL of 25 ng/µL were sent for 20 of the samples.

### Customization of microarray

Genotyping was performed with the Illumina Infinium Global Screening Array-24+ v3.0 Beadchip. The chip has 654,027 markers and the capacity for 100,000 additional custom markers. For this investigation, a limited number of custom markers were added from single

nucleotide variants (SNV) found to be associated with eGFRcrea in the study by Stanzick et al.[7] We added lead variants (n=424), index variants from independent signals derived from the identified loci (n=634) and variants included in credible sets with size <6 based on the independent signals (n=339). These additions comprised 1,066 unique SNVs. During the chip design process, additional studies of interest were published, and we added 1,460 SNVs from the study by Yu et al[8] and 59 SNVs from the study by Doke et al[S1]. After removal of overlap and duplicates these 2,585 Reference SNP cluster IDs (RSID) amounted to 2,370 unique RSIDs. Most of these were designed in triplicate. Of the 2,370 RSIDs with 2,669 SNVs in the custom design file, 2,320 (98%) and 2,438 (91%) were successfully incorporated on the chip as described in the SNV-table output from Genome Studio.

## Quality control

Quality control (QC) of the genotyping was performed by The Human Genomics Facility of the Genetic Laboratory of the Department of Internal Medicine at Erasmus MC. Cluster separation was good with no variants having a cluster separation lower than 0.27, which would indicate bad quality calls according to Illumina protocols. The minimal AB R mean, which represents the mean normalized intensity of the heterozygote cluster, was 0.21, meaning that all variants had good intensity values. The AB T mean, which measures if the heterozygote cluster has shifted towards the homozygote clusters, found 112 variants below 0.2 and 83 variants above 0.8, which were investigated more closely. After evaluation these variants were kept in the dataset as the clusters could be distinguished.

zCall was used to detect previously uncalled genotypes.[S2] Subsequently, another round of call rate and HWE filters were performed. No individuals were removed due to low call-rate (<99%) but one individual was removed due to excess of heterozygosity. Also, 797 SNVs

were removed due to low call-rate (<99%) and 23 SNVs failed a stringent (excess heterozygote) HWE filter ($p < 1*10^{-5}$).

## Genetic ancestry

Analyses of genetic ancestry were also performed by The Human Genomics Facility of the Genetic Laboratory of the Department of Internal Medicine at Erasmus MC. IBS/IBD distances between individuals and clustering with reference to the 2,504 samples of the 1000 Genomes Phase3v5 reference dataset (503 samples from European descent (EUR), 504 samples from East-Asian descent (EAS), 600 samples from African descent (AFR), 347 samples from Latin American descent (AMR), 489 samples from South-Asian descent (SAS) and 61 samples from African American descent) was performed.[6] The reference samples were merged with the study samples. To optimize the determination of ancestry, the 2,765 so-called Ancestry Informative Markers (AIMs) variants were used for this analysis. The IBS/IBD matrix was estimated using the --genome command of Plink. Multidimensional Scaling plots were produced (Supplementary Figure S2). Borders of the EUR samples were determined using the first 4 Principal Components (PCs) using a mean +/- 4SD approach. If the PCs of the study samples were bigger or smaller than the defined borders, the samples were defined as IBS outliers or non-European ancestry. In total, eight samples were identified as non-European. Since these few samples (0.5%) would have negligible effect on the results of the statistical analyses, they were not excluded from the dataset.

## Imputation

The number of SNVs imputed was 40,359,612, which is the expected number after using the HRC r1.1. There were 30,935,610 monomorphic SNVs, defined as a SNV having minor allele frequency (MAF) <=0.5%. The mean Markov Chain Haplotyping (MACH) $R^2$ for the whole dataset was 0.36, standard deviation (SD) 0.43, median 0.03. The percentages of SNVs with

MACH $R^2 > 0.8$ according to MAF were 11 (MAF<0.5%), 79 (MAF 0.5%-1%), 91 (MAF 1%-5%) and 98 (MAF>5%). MACH $R^2$ when excluding variants with MAF < 1% was 0.95 (SD 0.10). We used the Genome Reference Consortium Human Build 37 (Grch37) for SNV chromosomal positions.

# Supplementary Table S1

Table S1. Akaike Information Criterion for GAMMs of bias phenotypes with and without random effects. The Renal Iohexol Clearance Survey (RENIS).

| Dependent variable in GAMM | No random effects | Random intercept | Random intercept and slope |
|---|---|---|---|
| mGFR-eGFRcr | 9639.64 | 8800.45 | 8745.82 |
| mGFR-eGFRcys | 9120.20 | 8339.55 | 8295.71 |
| mGFR-eGFRcr-cys | 8237.99 | 7623.04 | 7627.04 |

GAMM, generalized additive mixed model.
Each GAMM was adjusted for sex-specific non-linear terms for time and sex-specific baseline age; and for sex, the first 10 principal components and their interactions with time.

## Supplementary Table S2

Table S2. Shapiro-Wilk tests of normality for original and log-transformed GFR phenotypes. The Renal Iohexol Clearance Survey (RENIS).

| Phenotype | Shapiro-Wilk statistic | P-value |
|---|---|---|
| mGFR phenotype | 0.99634 | 0.001 |
| eGFRcr phenotype | 0.93628 | 8.9069E-25 |
| eGFRcys phenotype | 0.95122 | 7.2783E-22 |
| eGFRcr-cys phenotype | 0.97342 | 5.7093E-16 |
| log(mGFR phenotype) | 0.97115 | 1.0418E-16 |
| log(eGFRcr phenotype) | 0.67130 | 6.3107E-47 |
| log(eGFRcys phenotype) | 0.88916 | 1.9859E-31 |
| log(eGFRcr-cys phenotype) | 0.88683 | 1.0736E-31 |

A constant of 5 was added before log-transformation.

## Supplementary Figure S1

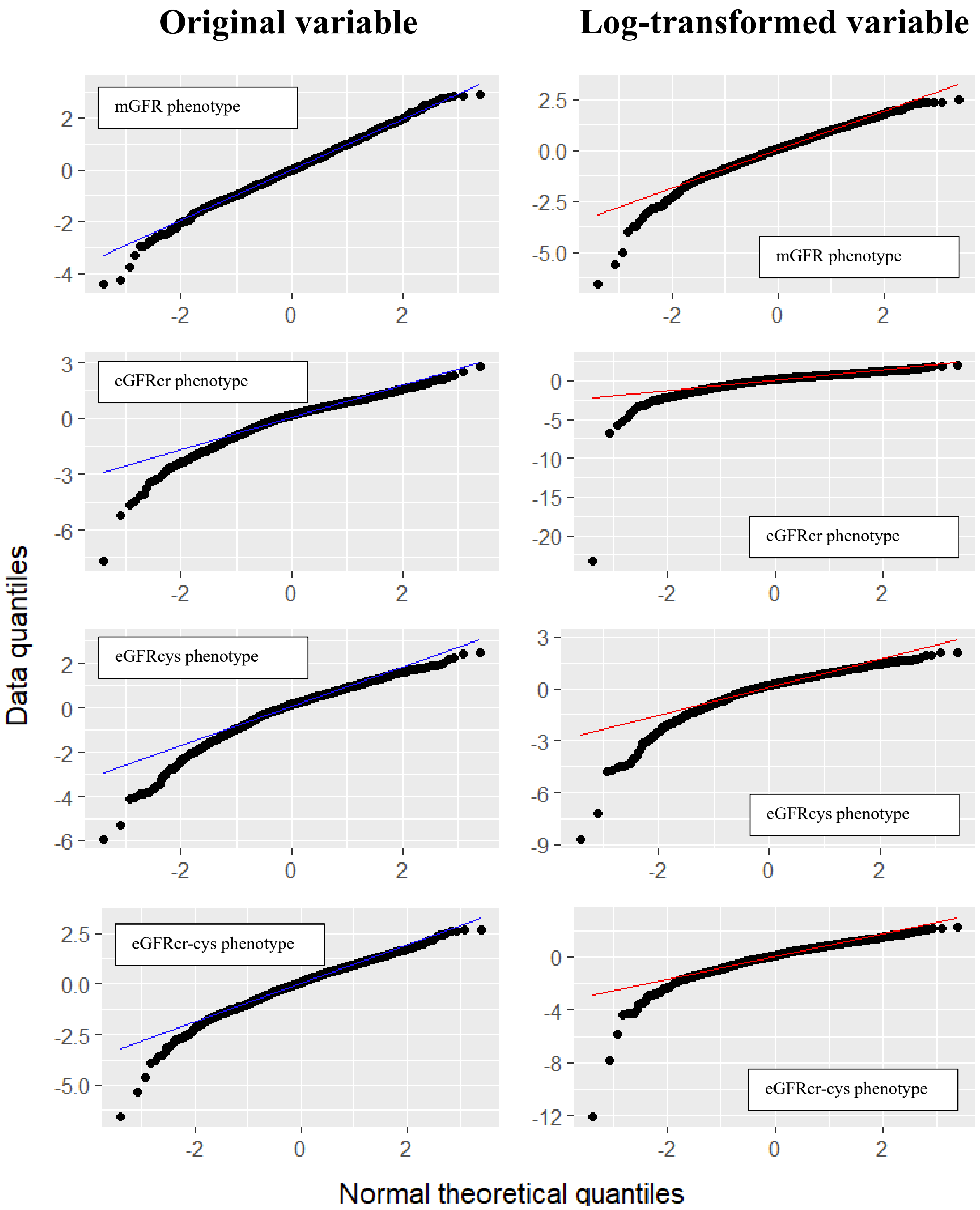


Figure S1. Q-Q plots for original and log-transformed versions of the GFR phenotypes. A constant of 5 was added before log-transformation.

# Supplementary Figure S2

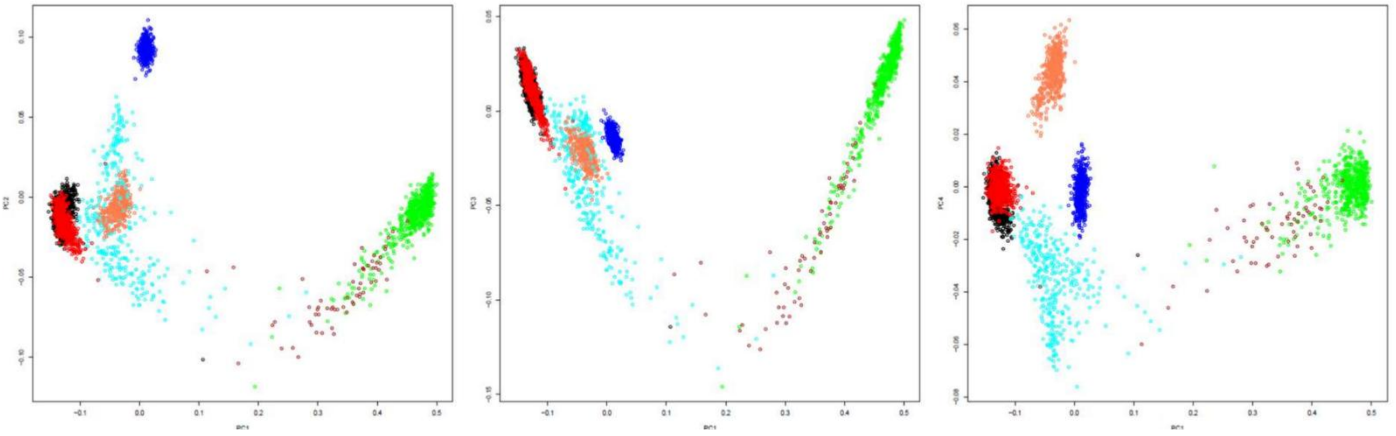

Figure S2. Principal Component Analysis (PCA). We used PCA to identify samples with another genetic ancestry than EUR. In Figure 2A, 2B, and 2C, the 4 different Principal Components (PC1, PC2, PC3, and PC4) were plotted. Red circles represent EUR samples, blue samples represent EAS samples, green circles represent AFR samples, cyan circles represent AMR samples, coral circles represent SAS samples, and brown circles represent African American samples. The black circles represent samples from the study population.

# Supplementary References


S1. Doke T, Huang S, Qiu C*, et al.* Transcriptome-wide association analysis identifies DACH1 as a kidney disease risk gene that contributes to fibrosis. *J Clin Invest* 2021; **131**.

S2. Goldstein JI, Crenshaw A, Carey J*, et al.* zCall: a rare variant caller for array-based genotyping: genetics and population analysis. *Bioinformatics* 2012; **28:** 2543-2545.

## STROBE Statement—checklist of items that should be included in reports of observational studies

| | Item No. | Recommendation | Page No. | Relevant text from manuscript |
|---|---|---|---|---|
| **Title and abstract** | 1 | (*a*) Indicate the study's design with a commonly used term in the title or the abstract | 1 | |
| | | (*b*) Provide in the abstract an informative and balanced summary of what was done and what was found | 2 | |
| **Introduction** | | | | |
| Background/rationale | 2 | Explain the scientific background and rationale for the investigation being reported | 4 | |
| Objectives | 3 | State specific objectives, including any prespecified hypotheses | 5 | |
| **Methods** | | | | |
| Study design | 4 | Present key elements of study design early in the paper | 6 | |
| Setting | 5 | Describe the setting, locations, and relevant dates, including periods of recruitment, exposure, follow-up, and data collection | 6 | |
| Participants | 6 | (*a*) *Cohort study*—Give the eligibility criteria, and the sources and methods of selection of participants. Describe methods of follow-up<br>*Case-control study*—Give the eligibility criteria, and the sources and methods of case ascertainment and control selection. Give the rationale for the choice of cases and controls<br>*Cross-sectional study*—Give the eligibility criteria, and the sources and methods of selection of participants | 6 | |
| | | (*b*) *Cohort study*—For matched studies, give matching criteria and number of exposed and unexposed<br>*Case-control study*—For matched studies, give matching criteria and the number of controls per case | | |
| Variables | 7 | Clearly define all outcomes, exposures, predictors, potential confounders, and effect modifiers. Give diagnostic criteria, if applicable | 6 - 13 | |

| | | | |
|---|---|---|---|
| Data sources/ measurement | 8* | For each variable of interest, give sources of data and details of methods of assessment (measurement). Describe comparability of assessment methods if there is more than one group | 6 - 13 |
| Bias | 9 | Describe any efforts to address potential sources of bias | 6-15 |
| Study size | 10 | Explain how the study size was arrived at | 6 |

| | | | |
|---|---|---|---|
| Quantitative variables | 11 | Explain how quantitative variables were handled in the analyses. If applicable, describe which groupings were chosen and why | 6-13 |
| Statistical methods | 12 | (*a*) Describe all statistical methods, including those used to control for confounding | 13-15 |
| | | (*b*) Describe any methods used to examine subgroups and interactions | |
| | | (*c*) Explain how missing data were addressed | 11 |
| | | (*d*) *Cohort study*—If applicable, explain how loss to follow-up was addressed<br>*Case-control study*—If applicable, explain how matching of cases and controls was addressed<br>*Cross-sectional study*—If applicable, describe analytical methods taking account of sampling strategy | 6 |
| | | (*e*) Describe any sensitivity analyses | |
| **Results** | | | |
| Participants | 13* | (a) Report numbers of individuals at each stage of study—eg numbers potentially eligible, examined for eligibility, confirmed eligible, included in the study, completing follow-up, and analysed | 6 |
| | | (b) Give reasons for non-participation at each stage | 6 |
| | | (c) Consider use of a flow diagram | Fig 1 |
| Descriptive data | 14* | (a) Give characteristics of study participants (eg demographic, clinical, social) and information on exposures and potential confounders | Table 1 |
| | | (b) Indicate number of participants with missing data for each variable of interest | |
| | | (c) *Cohort study*—Summarise follow-up time (eg, average and total amount) | 16 |
| Outcome data | 15* | *Cohort study*—Report numbers of outcome events or summary measures over time | 16 |
| | | *Case-control study*—Report numbers in each exposure category, or summary measures of exposure | |
| | | *Cross-sectional study*—Report numbers of outcome events or summary measures | |

| | | | |
|---|---|---|---|
| Main results | 16 | (*a*) Give unadjusted estimates and, if applicable, confounder-adjusted estimates and their precision (eg, 95% confidence interval). Make clear which confounders were adjusted for and why they were included | Table 3 |
| | | (*b*) Report category boundaries when continuous variables were categorized | n/a |
| | | (*c*) If relevant, consider translating estimates of relative risk into absolute risk for a meaningful time period | |

Continued on next page

| | | | |
|---|---|---|---|
| Other analyses | 17 | Report other analyses done—eg analyses of subgroups and interactions, and sensitivity analyses | |
| **Discussion** | | | |
| Key results | 18 | Summarise key results with reference to study objectives | 18 |
| | 19 | Discuss limitations of the study, taking into account sources of potential bias or imprecision. Discuss both direction and magnitude of any potential bias | 19 |
| Interpretation | 20 | Give a cautious overall interpretation of results considering objectives, limitations, multiplicity of analyses, results from similar studies, and other relevant evidence | 20 |
| Generalisability | 21 | Discuss the generalisability (external validity) of the study results | 19 |
| **Other information** | | | |
| Funding | 22 | Give the source of funding and the role of the funders for the present study and, if applicable, for the original study on which the present article is based | 21 |

*Give information separately for cases and controls in case-control studies and, if applicable, for exposed and unexposed groups in cohort and cross-sectional studies.

**Note:** An Explanation and Elaboration article discusses each checklist item and gives methodological background and published examples of transparent reporting. The STROBE checklist is best used in conjunction with this article (freely available on the Web sites of PLoS Medicine at http://www.plosmedicine.org/, Annals of Internal Medicine at http://www.annals.org/, and Epidemiology at http://www.epidem.com/). Information on the STROBE Initiative is available at www.strobe-statement.org.
